# Identification, Culture and Targeting of Cancer Stem Cells

**DOI:** 10.3390/life12020184

**Published:** 2022-01-27

**Authors:** Alejandro Herreros-Pomares

**Affiliations:** 1 Fundación de Investigación Hospital General Universitario de Valencia, 46014 Valencia, Spain; herreros_ale@gva.es or alherpo@etsiamn.upv.es; 2 Centro de Investigación Biomédica en Red Cáncer, CIBERONC, 28029 Madrid, Spain

**Keywords:** cancer stem cells, CSC makers, notch pathway, Wnt pathway, hedgehog pathway, Hippo pathway, cell culture, CSC culture

## Abstract

Chemoresistance, tumor progression, and metastasis are features that are frequently seen in cancer that have been associated with cancer stem cells (CSCs). These cells are a promising target in the future of cancer therapy but remain largely unknown. Deregulation of pathways that govern stemness in non-tumorigenic stem cells (SCs), such as Notch, Wnt, and Hedgehog pathways, has been described in CSC pathogenesis, but it is necessary to conduct further studies to discover potential new therapeutic targets. In addition, some markers for the identification and characterization of CSCs have been suggested, but the search for specific CSC markers in many cancer types is still under development. In addition, methods for CSC cultivation are also under development, with great heterogeneity existing in the protocols used. This review focuses on the most recent aspects of the identification, characterization, cultivation, and targeting of human CSCs, highlighting the advances achieved in the clinical implementation of therapies targeting CSCs and remarking those potential areas where more research is still required.

## 1. Tumor Heterogeneity: The Origin of the “Cancer Stem Cell” Concept

Cancers occur in an extraordinary range of types and subtypes, making each cancer individually unique [1]. Genetic and phenotypic differences are detected between tumors from different tissues and cell types, as well as between individuals with the same tumor type, a phenomenon known as inter-tumor heterogeneity [2]. In addition, they evolve differently over time in terms of clonal structure, genotype, and phenotype in every patient, complicating diagnosis, prognosis, and treatment [3]. Traditional pathology relies on phenotypic traits such as histological subtypes, treatment sensitivity, and clinical outcomes to classify tumors. However, genetic and phenotypic diversity occurs not only between tumors in different patients but also within populations of cells in a single tumor, which is called intra-tumor heterogeneity. Similar to inter-tumor heterogeneity, intra-tumor heterogeneity complicates diagnosis and obstructs therapeutic decision making. Firstly, spatial phenotypic heterogeneity can lead to a tissue biopsy that does not provide a reliable reflection of the whole tumor. Secondly, decision making based on scoring the dominant phenotype in a given specimen might be biased if they do not account for minor subpopulations with biologically and clinically relevant characteristics [4].

For these reasons, the analysis of tumor evolution is essential in addition to finding proper biomarkers to suitably distinguish tumor populations. Two major frameworks have emerged to explain cancer cell heterogeneity [5]. The clonal evolution model, also known as the stochastic model, was proposed by Nowell in 1976 and affirms that neoplasms start from a single cell of origin, and tumor progression results from acquired genetic variability within the original clone [6]. Consequently, the genetic and epigenetic variations that occur over time in individual cancer cells can confer a selective advantage in a Darwinian-like manner, permitting individual clones to create other clones leading to genetic heterogeneity and phenotypic and functional variations among the cancer cells within a single patient. In this model, the rate of cancer cells with tumorigenic potential is high, the tumor organization is not necessarily hierarchical, and the rational approach to therapy is targeting most or all cells [7,8]. 

The cancer stem cell (CSC) model, also called the deterministic model, emerged in the late 1990s when researchers began to address the possible relationship between hematopoietic stem cells and human leukemia [9,10]. It proposes that the growth and progression of numerous cancers are driven by small subpopulations of stem-like cells with self-renewal and differentiation features, named CSCs. In this model, the frequency of cancer cells with tumorigenic potential varies from rare to moderate, the tumor organization is always hierarchical, and the therapy approach enables targeting only tumorigenic cells. In particular, the CSC concept was coined in 1997, when Bonnet and Dick proved that human acute myeloid leukemia (AML) is organized as a hierarchy that originates from a primitive hematopoietic cell [11]. Since then, the presence of CSCs has been reported in a multitude of solid tumors, including the brain [12], breast [13], lung [14], colon [15], and pancreas [16]. In these tissues, it has been hypothesized that they arise from non-tumorigenic stem cells when a mutation that disturbs their ability to control cell division is generated. Stem cells are pluripotent and show self-renewal ability; therefore, CSCs might only use aberrantly stem cell pathways to support their proliferation. Alternatively, it was suggested that partially differentiated precursor cells, which are more frequent in adult tissue, could experience a few mutations that trigger their transformation to CSCs or that they could arise from differentiated cells that have undergone a de-differentiation process because of oncogenic mutations [17]. 

Even though these two models were considered mutually exclusive at first [18], clonal evolution and CSC models are now proposed as a unified model by some authors [19]. In the integrated model, the gaining of favorable mutations can result in clonal expansion of a founder cell. At some point, another cell may acquire new mutations that permit it to produce a new subclone. Over time, genetic mutations accumulate and subclones evolve concurrently. Here, CSCs are not considered static entities since they can evolve over a lifetime and genetic changes can influence their frequency in each subclone. Some subclones may present a marked hierarchical development, where few self-renewing CSCs exist among a larger number of bulk non-CSCs. Other subclones may contain an intermediate hierarchy, where the number of CSCs is relatively high and some other subclones may have genetic alterations that confer high-renewal potential, where most cells are tumorigenic [20].

**Figure 1:**
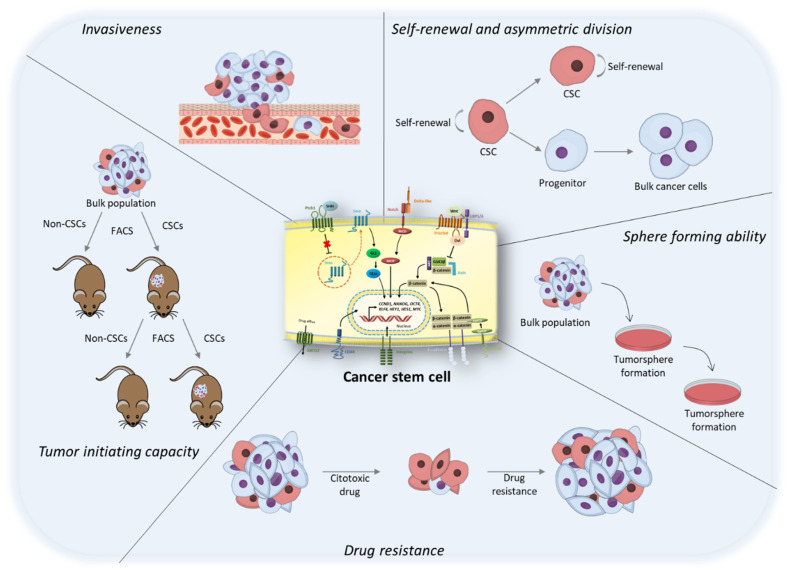
Main characteristics of cancer stem cells (CSCs). CSCs are able to generate daughter CSCs (self-renewal) and high proliferative bulk cancer populations by asymmetric cell division. In addition, they have the potential to proliferate indefinitely under anchorage-free conditions forming tumorspheres, a feature that has been linked to their invasion potential. Their quiescent slow-cycling phenotype together with the expression of certain molecules, such as efflux pumps, confers their drug resistance and the ability to form tumors when transplanted into animals, acting like tumor-initiating cells (TICs).

Although aberrantly regulated, CSCs share most of the mechanisms governing other stem cell populations (Figure 1). Firstly, they are able to generate daughter CSCs and high proliferative bulk cancer populations by asymmetric cell division, which is defined as any division that gives rise to two sister cells that have different fates—a feature that can be recognized by differences in size, morphology, gene expression pattern, or the number of subsequent cell divisions undergone by the two daughter cells [21]. CSCs have been described to undergo this type of division, maintaining their pluripotency by asymmetrically segregating their gene products into differentiating daughter cells [22]. Since CSCs have lost balance in networks regulating proto-oncogenes (promoting self-renewal), gate-keeping tumor suppressors (limiting self-renewal), and care-taking tumor suppressors (maintaining genomic integrity), they have the potential to proliferate indefinitely with minimal niche support. Soft agar formation and limiting dilution assays are commonly used to determine the frequency of self-renewing cells in a cell culture since CSCs can be serially transplanted through multiple generations [23].

Secondly, CSCs are known for their aberrant regulation of cell cycling, which permits them to display a quiescent slow-cycling phenotype tightly associated with treatment resistance and tumor dormancy [24]. Traditional chemotherapy regimens target proliferating cells, potentially missing their effect on slower dividing CSCs. This tumor cell population is able to enter into a reversible G0 phase from which cells may escape to reenter the cell cycle in response to physiological cell stimuli, such as cell death after treatment [25]. In this way, cell quiescence is not just a passive state but rather a condition actively maintained and regulated by signaling pathways allowing rapid activation of quiescent cells and reentry into the cell cycle [26]. In addition, protection of the stem cell population from damage or death is critical because these cells need to remain intact throughout the life of an organism. Ironically, it has been shown that efflux pumps also afford higher protection to CSCs, shielding them from the adverse effects of therapeutic agents [27]. ATP-binding cassette (ABC) transporters, including ABCG2, ABCB1, or ABCC1, to name but a few, are known to be associated with drug resistance in CSCs. A subset of CSCs that have a high capability for effluxing antimitotic drugs can be isolated by their capacity to efflux fluorescent dye Hoechst 33342 or rhodamine 123 with the help of a flow cytometer. This particular population is known as a side population (SP) because during flow cytometry analysis these cells can be visualized as a negatively stained population off to “the side” of the main cell population. The current understanding is that the drug-transporting capability of these cells is likely conferred by certain ABC transporters including ABCB1 (rhodamine 123) and ABCG2 (Hoechst 33342) [28].

Another interesting characteristic of CSCs is their anchorage-independent growth ability. Under anchorage-free conditions, the majority of cell types undergo anoikis, a specific type of cell death provoked by the loss of cell adhesion. Nevertheless, CSCs can grow independently of a solid surface [23]. It appears that cells that escape anoikis, represented mainly by stem cells and possibly early progenitor cells, synthesize higher levels of growth factors and extracellular matrix receptors, creating an in vitro niche that supports their survival and proliferation in suspension—being less dependent on cell–matrix and cell–cell interactions for survival [29]. In addition, CSC have been reported to overexpress antiapoptotic molecules, including BCL2 and BCLXL, which act as negative regulators of mitochondrial membrane permeabilization and cytochrome C release, survivin, belonging to the inhibitors of apoptosis (IAP) family members and lower levels of caspase 8 associated with tumor necrosis factor-α (TNF-α)-related apoptosis-inducing ligand (TRAIL) resistance [26]. 

The aforementioned characteristics are a direct result of the expression of signaling pathways related to self-renewal, proliferation, and differentiation, which are essential for stem cell populations [30,31]. The main difference between CSCs and other stem cell populations relies on their tumorigenic activity, given that CSCs are able to form tumors when transplanted into animals, acting like tumor-initiating cells (TICs), which is further discussed in the section: Culture of Cancer Stem Cells.

## 2. Cancer Stem Cells Markers and Pathways

Over the last decade, the evaluation of the expression profiles of cancer cells with stem properties in different solid tumors has allowed the identification of several biomarkers, pathways, and therapeutic targets against CSCs [32,33]. The main markers used for identifying and isolating CSCs include cell surface-adhesion molecules, cytoprotective enzymes, transcription factors, and drug efflux pumps [34]. However, CSCs markers in a determined organ or tissue are not completely shared with those markers that work in others, with a few of them shared between organs and tissues. Table 1 summarizes the molecules most frequently reported to act as CSC biomarkers.

**Figure 2:**
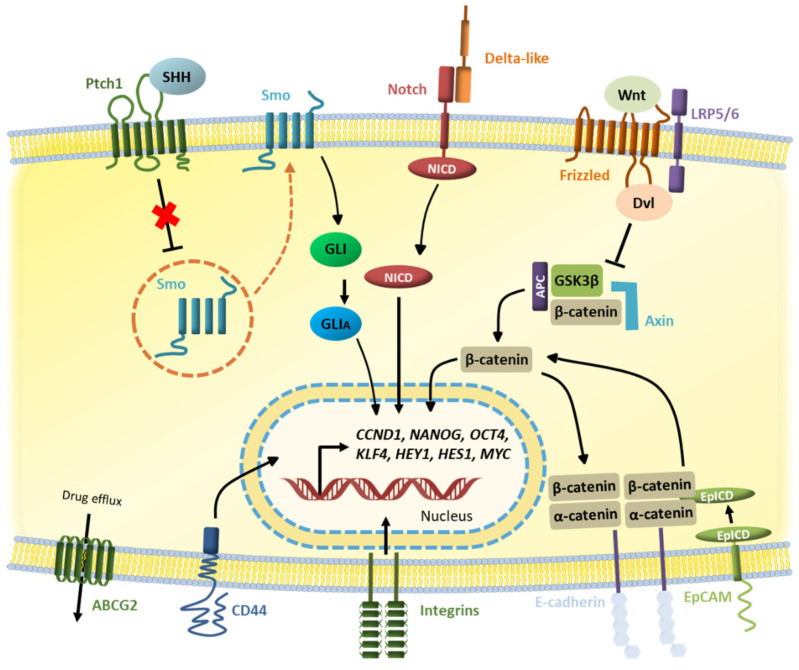
Core signaling pathways and molecules governing cancer stem cells. The expression of specific self-renewal, proliferation, and differentiation molecules and pathways confer CSCs their pathogenic characteristics.

In addition to the expression of specific self-renewal, proliferation, and differentiation molecules, CSCs have been reported to aberrantly express pathways that are essential for tissue development and homeostasis [30]. These pathways include Notch, Wnt/β-catenin, Hedgehog, JAK/STAT, TGF-β, or Hippo and their deregulation represents a key event for CSC propagation and pathogenesis (Figure 2) [67].

### 2.1. Notch Pathway

It is an evolutionarily conserved signaling cascade that constitutes a critical component in the molecular circuits that regulate a broad range of events during embryonic and post-natal development, including border formation, cell fate decisions, differentiation, migration, proliferation, and apoptosis [68,69]. The role of Notch in human malignancies has been recently highlighted by the presence of gain-of-function mutations and amplifications of Notch genes in different types of cancer, and by the evidence that genes in the Notch cascade are potential therapeutic targets [70,71]. The core components of the Notch pathway comprise four transmembrane receptors (Notch1–Notch4) and five structurally similar ligands (delta-like-1, -3, -4, and Jagged-1, -2) [72]. The Notch signaling cascade is initiated by ligand–receptor interactions between two neighboring cells resulting in two successive proteolytic events as part of the activation mechanism [73]. The first cleavage is mediated by a metalloprotease of the ADAM family (TACE, tumor necrosis factor-α-converting enzyme) in close proximity to the extracellular side of the plasma membrane. The released extracellular domain is then transendocytosed by the ligand-expressing cell. The second one occurs within the transmembrane domain, mediated by a multi-protein complex, called γ-secretase, consisting of presenilin, nicastrin, APH1, and PEN2, which leads to the release of the Notch intracellular domain (NICD). Upon cleavage, NICD translocates to the nucleus where it forms a complex with the ubiquitously expressed transcription factor CBF1. The translocation of NICD is counteracted by Numb, through a mechanism that is not completely understood [74]. In the absence of NICD, CBF1 is a transcriptional repressor due to its association with co-repressors. When NICD associates with CBF1, a number of co-activators are recruited, including mastermind-like (MAML) -1, -2, and -3, resulting in a multiprotein complex that acts as a potent transcriptional activator. The most well-defined targets of the NICD-CBF1 complex are the hairy enhancer of the split (HES) family, the Hes-related repressor protein (HERP, also called HEY) family, cell cycle regulators, such as *CDKN1A* and *CCND1*, and apoptosis regulators [75,76].

The first link between Notch and human tumors was made in the late 1980s in a small number of patients suffering from T cell acute lymphoblastic leukemia (T-ALL) [77]. More recently, deregulated expression of members of the Notch signaling pathway has also been reported in many solid tumors, including breast [78] and lung cancers [79]. Notch pathway function has been seen to be context dependent, since different Notch receptors or ligands could induce different gene expression programs, explaining the different and even opposite outcomes observed in this signaling pathway for different solid tumors. There are four major pleiotropic effects that the Notch pathway carries out and are relevant during tumorigenesis: (a) Gate-keeper function. Notch maintains stem cells in an undifferentiated state. In the intestine, for example, Notch prevents crypt progenitor cells from differentiating; (b) binary cell fate decisions: In the lymphoid system, it specifies the T cell lineage at the expense of the B cell lineage from a bi-potent early thymocyte progenitor; (c) induction of differentiation. In the skin, Notch induces terminal differentiation events, and during thymocyte differentiation, Notch1 promotes differentiation of pro-T cells into pre-T cells. (d) Tumorigenesis. Overexpression of Notch within hematopoietic bone marrow cells or in T cell progenitors results in T-cell leukemias and as such, Notch functions as an oncogene. However, in the skin, Notch functions as a tumor repressor since the loss of Notch signaling results in the development of basal cell carcinoma-like tumors [80]. Notch pathway expression is not only tissue dependent, but also cell dependent. Moreover, while in SCLC Notch signaling is not active, in NSCLC it was found to be active, possibly due to the loss of Numb inhibitor expression or to the presence of gain-of-function mutations in Notch receptors [74], leading to high expression levels of Notch target genes and making this tumor type susceptible to therapies based on Notch inhibition [81]. The involvement of Notch on lung cancer was experimentally proven in a transgenic mouse model by the alveolar epithelium-specific expression of activated Notch. The mice developed alveolar hyperplasia as early as 7 days after *NOTCH1* induction with a Dox system and, when crossed with mice conditionally overexpressing *MYC* in the alveolar epithelium, mice developed adenocarcinomas [82].

### 2.2. Wnt Pathway

The evolutionarily conserved Wingless-type protein (Wnt) signaling pathway plays an important role in controlling a number of embryonic development processes and the maintenance of tissue homeostasis in adults by regulating proliferation, differentiation, migration and polarity, survival, genomic stability, and self-renewal of stem cells [83]. Not surprisingly, aberrant Wnt signaling underlies a wide range of diseases, including cancer [84], fibrosis [85], and neurodegenerative disorders [86]. The Wnt signaling network is complex. Firstly, there are 19 Wnt ligands, which are glycoproteins of 40 kDa in size that contain lipid modifications with many conserved cysteines, and more than 15 receptors and co-receptors distributed over seven protein families in mammals [87]. Moreover, Wnt proteins can trigger a variety of responses, often gathered at two groups: the canonical Wnt signaling pathway, for the classical Wnt-induced activation of β-catenin-TCF (T-cell factor) transcriptional complexes, and the non-canonical Wnt signaling pathway, which includes the planar cell polarity (PCP) signaling pathway [88], the Wnt/Ca^++^ flux pathway and the protein kinase A pathway [89,90] and cJun N-terminal kinase (JNK) and small GTPase Rho, Rac, and Cdc 42 signaling networks [91,92]. Moreover, crosstalk from various non-Wnt factors has also been reported to modulate nuclear β-catenin accumulation [93]. 

Given the importance of WNT signaling for adult stem cell biology, it is not surprising that WNT pathway mutations are frequently observed in cancer. A role for the WNT pathway in cancer was first described in the 1980s and 1990s in mouse models of mammary cancer and in human and mouse colon cancer. Researchers found that the induction of an aberrant overexpression of *WNT1* using a proviral insertion at its locus or by transgenesis generated breast tumors in mice [94]. Other studies suggested a critical role of *WNT-CTNNB1* signaling in colorectal cancer [95,96]. Germline-inactivating mutations in the *APC* gene, which is a negative regulator of *CTNNB1* (gene encoding for β-catenin), were found in patients with a hereditary cancer syndrome termed familial adenomatous polyposis (FAP), which can progress to colorectal carcinomas following concomitant activating mutations in *KRAS* and inactivating mutations in *TP53* [97]. Both the *APC* gene and *CTNNB1* are often mutated in colorectal cancers of non-FAP patients, and overexpression of constitutively active *CTNBB1* or loss of APC function can result in colorectal tumorigenesis [84]. Finally, it has been reported that the Wnt signaling pathway helps to maintain the CSCs population in lung cancer. Increased expression of *CTNNB1* has been associated with the overexpression of putative stem cells markers, such as CD44, EPCAM, OCT4, and CCND1, and resistance to a number of chemotherapeutic drugs in sorted lung CSCs [98]. In addition, Nakashima et al. found that WNT3 promotes tumor progression in a study including 128 resected NSCLC patients [99]. 

### 2.3. Hedgehog Pathway

The hedgehog (Hh) signaling pathway is an important component in the regulation of stem cell properties during embryonic development and in adult tissues. During embryogenesis, it controls proliferation and differentiation in a time- and position-dependent manner and plays a central role in tissue repair and regeneration in adult tissues. Mutations and deregulations of genes related to the Hh pathway have been reported in some solid tumors, contributing to the onset of cancer and accelerating the rate of tumor growth [100]. The mammalian Hh signaling pathway is mainly constituted by three Hh ligand homologs with different spatial and temporal distribution patterns, sonic hedgehog (SHH), Indian hedgehog (IHH), and desert hedgehog (DHH), two transmembrane receptors, patched homolog 1 and 2 (PTCH1, -2), a G protein-coupled receptor called smoothened (SMO), and a cytoplasmatic complex that regulates the glioma-associated oncogene homolog (GLI) family. GLI1 is a transcription activator, and GLI2 and GLI3 are both activators and repressors of transcription. The Hh signaling cascade is initiated by Hh ligands binding to the PTCH1 protein on the target cell. In the absence of Hh ligands, PTCH1 represses the activity of SMO, preventing its localization to the cell surface from intracellular endosomes, where SMO is predominantly located. Under these circumstances, different kinases phosphorylate and activate repressor forms of GLI transcription factors. The active form of GLI is prevented from transactivating Hh-responsive genes by the serine-threonine protein kinase suppressor of fused (SUFU) and the atypical kinesin-like protein Costa (COS) in a manner that is still not completely understood. Upon binding of the Hh ligand, PTCH1 is internalized and apparently destabilized, so that it can no longer transport the endogenous agonist molecules outwards. This allows them to accumulate intracellularly and activate SMO, which sequestrate COS and SUFU, releasing the GLI transcription factors to exert their effects in the nucleus. KIF3A and β-arrestin are required for SMO activation [101,102].

The first connection between aberrant Hh signaling and cancer was the discovery of a mutation in the transmembrane receptor *PTCH1* that causes a rare condition, named Gorlin syndrome [103]. Gorlin syndrome patients suffer from various basal cell carcinomas throughout their lifetimes and are predisposed towards other types of cancer. Additionally, increased Hh signaling has been reported to be involved in a third of all human medulloblastoma cases, frequently due to *PTCH1* and *SUFU* mutations. In all these cases, it is believed that deregulated Hh signaling leads to increased cell proliferation and tumor formation [104,105]. Many malignancies have been linked to aberrant Hh signaling in CSCs, including oral and esophageal cancers [106]. 

### 2.4. Hippo Pathway

This signaling pathway has emerged as an evolutionarily conserved regulator of diverse cellular processes, including cell survival, proliferation, and differentiation. Like the Notch, Wnt, and Hh pathways, the Hippo pathway plays a fundamental role in tissue homeostasis, organ size, and regeneration and its deregulation has been associated with tumorigenesis in several malignancies, including breast [107] or oral [108] cancers. The central components of the Hippo pathway in mammals are well defined, but most of the upstream regulators of this pathway remain unknown. Essentially, it consists of an inhibitory serine/threonine kinase module and a transcriptional module. The first one is composed of the mammalian Ste20-like protein kinase 1 and 2, (MST1 and -2), the large tumor suppressor kinase 1 and 2 (LATS1 and -2), and the activating adaptor proteins: Salvador family WW domain-containing protein 1 (SAV1) and the MOB kinase activator 1A and 1B (MOB1A and -B). The transcriptional module comprises the transcriptional co-activators yes-associated protein (YAP), the transcriptional co-activator with PDZ-binding motif (TAZ), which is a paralog of YAP, and the TEA domain family members (TEAD1–TEAD4). When Hippo signaling is activated, LATS1 and LATS2 phosphorylate YAP and TAZ, resulting in YAP/TAZ sequestration in the cytoplasm via binding to 14-3-3 and degradation in a ubiquitin–proteasome-dependent manner. When YAP/TAZ are not phosphorylated by LATS kinases, they translocate to the nucleus and bind to sequence-specific transcription factors, including TEAD family, Smad, Runx1/2, p73, ErbB4, Pax3, and T-box transcription factor 5 (TBX5) to mediate the transcription of target genes encoding proteins that are involved in cell proliferation and survival, such as angiomotin-like protein 2 (AMOTL2), amphiregulin (AREG), baculoviral IAP repeat-containing protein 5 (BIRC5), connective tissue growth factor (CTGF), and cysteine-rich angiogenic inducer 61 (CYR61) [109,110,111,112]. 

Silencing of components from the inhibitory module and overexpression of those from the transcriptional module of the Hippo pathway has been associated with CSCs phenotype. Long-term activation of YAP/TAZ results in cell transformation and has been correlated with several human cancers, including liver, lung, colorectal, ovarian, and prostate [113,114]. For instance, TAZ activation conferred CSC-related traits on breast cancer cells [107] and YAP was shown to occupy mammary stem cell signature gene promoters to induce breast CSCs [115]. In addition, glucocorticoid hormone-induced YAP activation has been reported to expand chemoresistant breast CSCs [116]. Similarly, overexpression of YAP protein was associated with progression and poor prognosis of NSCLC in an immunohistochemistry study including 92 patients [117] and different in vitro and in vivo experiments have associated YAP/TAZ expression with cellular migration, metastasis, and tumor growth [118,119]. Finally, YAP is also a major inducer of CSC properties by direct upregulation of SOX2 and SOX9, maintaining CSCs in esophageal cancer, osteosarcoma and glioblastoma [120,121]. On the contrary, MST1 and -2 along with LATS1 and -2 have been defined as tumor suppressors in mice and inactivating mutations on LATS2 have been found in nearly 40% of human mesothelioma tumors [122,123]. In that sense, *MST1* overexpression was found to inhibit the growth of NSCLC cells in vitro and in vivo, having an antiproliferative effect associated with the induction of apoptosis [124]. 

### 2.5. JAK/STAT Pathway

The activation of this pathway has been associated with an increased expression of adhesion molecules, such as intercellular cell adhesion molecule (ICAM) and vascular cell adhesion molecule (VCAM), which has been associated in turn with growth and survival in cancer cells, tumor progression and metastasis. For instance, human prostate CD133^high^/CD44^high^ CSCs exhibited ICAM-1 and VCAM-1 immunoreaction compared with non-CSCs [125]. In addition, STAT3–NFkB signaling is known to be activated in breast CSCs and the inhibition of STAT3 in two breast cancer cell lines reduced CD44^+^ CSC marker cell population [126]. In lung cancer, STAT3 is known to function as a transcription factor with target genes that are important for cell proliferation, induction of angiogenesis, prevention of apoptosis, evasion of host immune surveillance, and CSCs renewal [127].

Altogether, the data obtained so far indicate that CSCs are tightly linked to patient outcomes, being an important tumor population to be addressed. Identifying molecular alterations that could act as potential targets and biomarkers against this tumor population could have major implications in NSCLC management. Therefore, it is important to continue characterizing CSCs to better understand their role in treatment resistance, relapse, and metastasis associated with the high mortality that is typical of lung cancer.

## 3. Culture of Cancer Stem Cells

Since stem cells display anchorage-independent growth ability, sphere-forming assays have become the gold standard in their cultivation as a method for their isolation and enrichment [128]. These assays consist of culturing cells under non-adherent conditions using serum-free medium supplemented with minimum growing requirements using fresh tumor tissue or commercial cell lines as starting material. Using cells directly isolated from surgical resections specimens can reflect in vivo conditions better than cell lines, but the establishment of primary cultures is problematic and time consuming, mainly because of the frequent lack of cell viability, the excessive necrosis of some tumor samples, and the proliferation of non-tumorigenic cell types in cultures. As a result, most studies to date have been performed in commercially available cell lines. Sphere-forming assays were first used to culture cells from the adult brain, obtaining stem-like cells as free-floating spheres, called neurospheres [129]. Since then, these culture conditions have been widely used to evaluated self-renewal and differentiation at the single-cell level in vitro, assuming a relevant role to identify CSCs due to the general lack of unique CSCs markers and the absence of distinctive morphological phenotypes [130]. Several studies have reported the identification of cells with stem cell properties in cell lines and primary cultures established from cancer patients [131,132,133,134,135,136,137,138]. However, most publications display substantial variations in different aspects of sphere-forming assays, including changes in medium composition and volume, cell density, the surface area of the culture dish, and the duration in culture before quantification [139,140]. This diversity in procedures has promoted differing and even opposing results to arise from some groups. Results range from those who argue that the formation of tumorspheres with increased stemness can be performed without the addition of any external mitogenic stimulation [141] to those who reported that tumorspheres cultivation requires exogenous mitogens supplementation, including epidermal growth factor (EGF), basic fibroblast growth factor (bFGF), insulin-transferrin-sodium selenite, B27 or hydrocortisone [142,143]. Moreover, high variability in the kinetics of the formation of tumorspheres between cell lines and between primary cultures from patient samples has also been detected, making it even more difficult to know if reported variations are due to changes in culture conditions or due to the CSC population of the cell line/patient cultured. Another determinant parameter of sphere-forming assays is cell density, given that it plays a critical role in clonality. Initially, these assays were conceived to obtain clonal spheres formed from one single cell. For that aim, cells are normally plated at extremely low densities since high seeding cell densities can make the interpretation of results difficult due to the fusion of spheres. However, tumorspheres are highly dynamic entities and have been observed to frequently aggregate and fuse, even at low densities, so that true clonality can only be assured by plating single cells per well. However, single-cell plating also has a negative impact on cell viability due to the lack of autocrine/paracrine signals released by cells into the medium and can be extremely challenging in some cases [144,145,146]. 

For all of the above reasons, some authors have cast doubts on the premise that sphere-forming assays allow distinguishing in vivo stem cells, considering that these in vitro assays evaluate actually the ability of cells to act as stem cells when are taken from its in vivo niche [147]. In fact, sphere-forming assays allow determining ex vivo proliferation, but they cannot evaluate the ability of CSCs to initiate nor propagate tumors. To overcome these limitations, the usage of transplantation assays in animal models is widely extended [148,149,150]. In these in vivo assays, selected populations of tumor cells are transplanted into immunocompromised animals (usually NOD/SCID mice) in order to confirm the TIC capacity of these cells. It has been reported that a large number of tumor cells, in the order of millions of cells, are required to initiate tumor growth when xenotransplanted into animal models [151,152,153]. However, if sorted based on specific CSC markers, only millions of cancer cells are required to give rise to a whole tumor. Alternative culture conditions, such as the usage of scaffolds and other supporting materials, are also under study although their implementation is still limited [154].

## 4. Targeting of Cancer Stem Cells

Given their relevance in tumor development and maintenance, CSC markers and signaling pathways have been explored for over three decades as desirable targets. Notably, advances include the FDA approval of several molecules, but determining the best clinical trial design for investigating specific targeting of CSC remains challenging [155,156,157].

For CSC markers, monoclonal antibodies targeting EpCAM have received special attention in clinical trials [158]. Edrecolomab was the first EpCAM targeting test in patients, showing a significant increase in the OS of colorectal cancer patients [159,160]. However, subsequent larger studies could not confirm its clinical activity [161,162,163]. Three other clinically tested anti-EpCAM monoclonal antibodies with different targets have been designed: 3622W94, ING-1, and the fully human adecatumumab. Remarkably, acute pancreatitis cases were detected after treatment with the highest affinity antibodies (3622W94 and ING-1), even at low concentrations (1 mg/kg) [164]. On the contrary, several phase I and phase II studies using adecatumumab in hormone-resistant prostate cancer patients reported minimal secondary effects and great clinical potential [165,166,167,168]. Catumaxomab, a hybrid mouse IgG2a/rat IgG2b antibody that is trispecific for EpCAM, CD3, and, via the Fcc receptor, that activates accessory cells such as macrophages, NK cells and DCs, obtained market approval in Europe in 2009 to treat malignant ascites in cancer patients. Most patients develop a tolerable humoral response against catumaxomab due to the presence of the chimeric Fcc domain, which evokes an immunogenic reaction that correlates with improved overall survival and controllable side effects [169]. EpCAM-specific immunotoxins are also being used in clinical trials. A phase I/II study with proxinium, a promising humanized anti-EpCAM antibody with a *Pseudomonas* exotoxin, provided 88% of stable diseases or responses in local head and neck cancers, 25% of them with a complete remission of the disease [170]. The same antibody construct, named Vicinium for use in bladder cancer, showed satisfactory results in a Phase II study in patients with urothelial carcinoma in situ of the bladder, where 44% of patients achieved a complete response after treatment with 30 mg/dose for 6–12 weeks of intravesical vicinium [171]. However, most ongoing clinical trials are focused on determining the safety and efficacy of CAR-T cells by recognizing EpCAM (Table 2). Two phase II studies are currently recruiting patients with relapsed or refractory liver (NCT02729493) and stomach (NCT02725125) cancers to evaluate the efficacy and safety of EpCAM-targeted CAR-T cells. Another phase I study (NCT02915445) in EpCAM+ recurrent or refractory nasopharyngeal carcinoma and breast cancer patients, and a phase I/II study (NCT03013712) for EpCAM-positive cancers (colon, esophageal, pancreatic, prostate, gastric, or hepatic carcinomas) are also ongoing, which indicates the great potential that CAR-T cell therapy can have in cancer treatment.

Regarding other CSC markers, although numerous preclinical studies have shown promising results for CD44, clinical trials have shown a lack of efficacy or fatal toxicity. A phase I trial involving patients with advanced-stage solid tumors revealed that the humanized anti-CD44 RG7356 has limited clinical efficacy with 21% of patients showing stable disease [172]. On the other hand, bivatuzumab mertansine was found to cause deadly skin toxicity to one patient with head and neck squamous cell carcinoma in a phase I trial [173]. Again, most ongoing clinical trials involving CD44 are focused on determining the safety and efficacy of CAR-T cells (Table 2). For CD133, a phase I study of CAR-T cells targeting CD133 in 23 patients with advanced-stage hepatocellular, pancreatic, or colorectal carcinoma revealed a manageable toxicity profile, mainly with grade ≤ 3 decreases in hemoglobin levels and/or platelet counts that recovered rapidly and spontaneously, as well as signs of efficacy, including three partial responses [174]. Likewise, a phase I trial to determine the safety and efficacy of CAR-T cells in patients with recurrent malignant gliomas with the expression of CD133 is currently ongoing, whereas a phase II clinical trial with a CD166-directed probody drug conjugate (PDC), CX-2009, is currently recruiting patients with advanced, metastatic breast cancer. Finally, several clinical trials are targeting integrins using monoclonal antibodies, small molecules, and cytotoxins (Table 2).

For CSC-related pathways, considerable progress has been achieved in clinical trials for Notch and Hh pathway inhibitors. There are two major methods used to inhibit Notch signaling: γ-secretase inhibitors (GSIs) and anti-Notch receptor or ligand antibodies [175]. MK-0752 was the first GSI used to treat T-ALL in children in a phase I trial with poor results [176]. The lack of clinical activity was confirmed in subsequent phase II trials: only one patient suffering from interdegenerative astrocytoma had a complete response out of 10 patients with different types of glioma [177]. On the contrary, combining MK-0752 with cisplatin treatment for ovarian cancer, docetaxel treatment for locally advanced or metastatic breast cancer, and gemcitabine treatment for ductal adenocarcinoma of the pancreas (PDAC) has shown better efficacy [178,179,180]. For instance, the combination of MK-0752 with gemcitabine in first- or second-line treatment of PDAC achieved 13 out of 19 stable diseases (68.4%) and one partial response [180]. However, the clinical effect has been proved to be minimal in patients with advanced solid tumors, including pancreatic cancer [181,182]. RO4929097 is another selective GSI that has shown good anti-tumor activity in preclinical and early clinical trials [183,184]. Combinations of RO4929097 with gemcitabine, temsirolimus, or cediranib in advanced solid tumors, as well as with bevacizumab in recurrent high-grade glioma have shown controllable toxicities with moderate clinical benefit [185,186]. However, the combination of RO4929097 with vismodegib showed no significant differences for recurrent platinum-resistant ovarian cancer, metastatic colorectal cancer, and pancreatic adenocarcinoma [187,188,189]. Similar results have been obtained for the GSI PF-03084014. In a phase I trial involving 64 advanced-stage solid tumors [190], one patient with thyroid cancer had a complete response and five out of seven with desmoid tumors exhibited partial responses. In addition, a phase II trial including patients with refractory desmoid tumors reported partial responses in five out of 16 patients and five prolonged stable diseases with controlled side effects and significant improvements in performance status [191]. Remarkably, three out of five patients who achieved objective responses did so almost 2 years after the start of treatment, suggesting a mechanism of action distinct from that of standard cytotoxic therapies. Finally, in a phase I trial including patients with T-ALL and T cell lymphoblastic lymphoma, PF-03084014 treatment achieved a complete response in a heavily pre-treated patient with T-ALL harboring an activating NOTCH1 mutation (L1679P) [192]. Encouraging findings have also been obtained with the GSI BMS-906024 [193]. In a phase I study including 25 patients with relapsed or refractory T-ALL or T cell lymphoblastic lymphoma, eight patients showed a more than 50% reduction in their bone marrow blast counts with responses occurring in both NOTCH1-mutant and NOTCH1-wild-type subgroups. Other selective GSIs, such as BMS-986115 (NCT01986218), CB103 (NCT03422679), LY3039478 (NCT02836600), and LY900009 (NCT01158404) are also under investigation in clinical trials with results pending publication. Targeting the DLL4 ligand is another approach to block Notch signaling which is being tested. Demcizumab, a humanized IgG2 monoclonal antibody, was tested in a phase I trial involving 55 patients with previously treated solid tumors and showing promising results [194]. The DENALI study, a phase Ib clinical trial combining demcizumab with carboplatin and pemetrexed to treat non-squamous non-small cell lung cancer (NSCLC), found objective responses in half of the patients [155]. However, the addition of demcizumab to gemcitabine and nab-paclitaxel in the first-line treatment of patients with metastatic PDAC did not improve efficacy (NCT02289898). Enoticumab, another fully human IgG1 antibody against DLL4, has also shown promising activity for advanced solid tumors. In a phase I study including patients with different advanced solid malignancies, enoticumab achieved two partial responses and 16 stable diseases in 44 patients with manageable safety profiles [195]. 

Regarding Hh signaling, several SMO antagonists have been tested in clinical trials, resulting in regulatory approvals for the treatment of basal cell carcinoma (BCC) and AML. Vismodegib was the first Hh pathway inhibitor approved by the FDA in 2012 for the treatment of locally advanced or metastatic BCC, with overall response rates of 60.3% and 48.5%, respectively [196]. In the following international open-label phase II trial, STEVIE, locally advanced BCC had a response rate of 68.5%, whereas the response rate of metastatic BCC was 36.9% [197]. Subsequent phase I and II trials targeting recurrent medulloblastoma have shown that the progression-free survival (PFS) of adult and pediatric patients with SHH-subtype medulloblastoma treated with vismodegib is longer [198,199]. Unfortunately, the treatment of chondrosarcoma, metastatic colorectal, pancreatic, gastric, or ovarian cancers with vismodegib has not resulted in better survival [200,201,202,203,204]. In parallel, sonidegib was approved by the FDA in 2015 for patients with locally advanced BCC that recurred and are not suitable for surgery or radiation therapy [205]. A phase II trial for patients with recurrent medulloblastoma with sonidegib plus cisplatin and etoposide resulted in complete or partial responses in half of the cases [206]. These combinations showed sustained PFS in small-cell lung cancer patients with Sox2 amplification [207] and are under investigation in prostate (NCT02111187), breast (NCT02027376), and recurrent ovarian (NCT02195973) cancers. In 2018, glasdegib was approved in combination with low-dose cytarabine for the treatment of AML in patients older than 75 years or ineligible for intensive induction chemotherapy with a significant improvement in overall survival (OS) [208]. In an ongoing phase II trial, the addition of glasdegib to cytarabine/daunorubicin also showed a significant efficacy in patients with AML, chronic myeloid leukemia (CML), or high-risk myelodysplastic syndromes with manageable toxicity [209]. A phase III trial is also ongoing on the combination of glasdegib with standard induction therapy of AML (NCT03416179). Other selective SMO inhibitors have also entered clinical trials, including taladegib and saridegib. Taladegib showed an overall ORR of 46.8% in patients with advanced-stage BCC, whereas the combination of saridegib with cetuximab in head and neck squamous cell carcinoma patients has shown a more moderate efficacy [210,211].

## 5. Conclusions

In the current context of cancer research, CSCs have been discovered as promising targets with many questions to be solved. Many studies have been conducted in commercial cancer cell lines, but few data are available from patients’ samples. In many cancer types, there are no robust biomarkers available for CSCs identification. Therefore, it is necessary to invest in identifying CSCs markers that are able to discriminate CSCs in order to develop specific therapies against this population. Sphere-forming assays are a satisfactory method for CSCs isolation and enrichment, permitting to obtain suitable in vitro models. However, improvements in these in vitro assays are still required in order to avoid experimental variability to affect result interpretation and standardize the critical parameters of protocols used by researchers. Identification of potential targets against CSCs is a promising therapeutic strategy for the development of new drugs and the improvement of the cancer patients’ survival. Some of the targets already identified have proven satisfactory results, leading to market approvals that could change the future in the management of oncologic patients.

## Figures and Tables

**Table 1:** Compilation of frequently proposed CSCs markers for distinct tumor types.

Molecule	Tumor Type	Function	Refs
ABCG2	Liver, lung, melanoma, pancreatic	It is an ABC drug transporter that act as efflux pump to protect cells from xenobiotic toxins.	[35,36,37,38]
ALCAM (CD166)	Colorectal, head and neck, lung, pancreatic	A highly preserved transmembrane protein that belongs to the immunoglobulin superfamily. It binds to the T cell differentiation antigen CD6 and involves in cell adhesion and migration processes.	[34,39,40,41]
ALDH1	Breast, colorectal, lung, melanoma, ovarian, pancreatic, prostate	A group of NAD(P)+-dependent enzymes that catalyze the oxidization of aldehydes into carboxylic acids, playing a role in drug resistance.	[42,43,44,45,46,47,48]
CD133 (PROM1)	Breast, colorectal, glioma, liver, lung, melanoma, ovarian, pancreatic, prostate	A pentaspan transmembrane glycoprotein that maintains lipid composition in cell membranes. Evidence suggest that CD133+ cells display strong resistance to chemo-, radio- and immunotherapy.	[36,42,49,50,51,52,53,54]
CD44	Breast, colorectal, glioma, liver, lung, ovarian, pancreatic, prostate	A cell surface glycoprotein that acts as a receptor for many extracellular matrix components, including acid hyaluronic, collagen, integrins and metalloproteinases, promoting cell migration and self-renewal.	[43,51,55,56,57,58,59,60]
CD90 (THY1)	Breast, glioma, liver, lung	A highly conserved glycophosphatidylinositol (GPI)-anchored cell surface glycoprotein that participates in T cell adhesion and signal transduction.	[61]
EpCAM (CD326)	Colorectal, liver, lung, ovarian, prostate	A transmembraneglycoprotein expressed on most normal epithelial cells that acts as a homotypic calcium-independent cell adhesion molecule.	[44,62,63]
Integrin α6β4	Breast, colorectal, lung, prostate	A cellular adhesion molecule that binds to laminins in the extracellular matrix and nucleates the formation of hemidesmosomes, enabling cell migration and invasion	[56,64,65,66]

**Table 2:** CSCs marker-directed therapeutic approaches in clinical development.

Target	Therapeutic Strategy	Class	Ongoing Trial	Identifier	Current Status
EpCAM	Catumaxomab	Trispecific EpCAM/CD3/Fcc antibody	Phase II in gastric cancer with peritoneal carcinomatosis	NCT01504256	Completed
	Vicinium	Immunotoxin	Phase III in bladder cancer	NCT02449239	Active, not recruting
	EpCAM CAR-T	Autologous T cells engineered	Phase I in nasopharyngeal carcinoma and breast cancer	NCT02915445	Recruiting
	EpCAM CAR-T	Autologous T cells engineered	Phase I/II in colon, esophageal, pancreatic, prostate, gastric and hepatic cancer	NCT03013712	Unknown
	EpCAM CAR-T	Autologous T cells engineered	Phase II in liver cancer	NCT02729493	Unknown
	EpCAM CAR-T	Autologous T cells engineered	Phase II in gastric cancer	NCT02725125	Unknown
CD44	RO5429083	Anti-CD44 monoclonal antibody	Phase I in advanced CD44-expressing malignant solid tumors	NCT01358903	Completed
	SPL-108	CD44 targeted agent	Phase I in ovarian epithelial cancer	NCT03078400	Active, not recruting
	AMC303	CD44v6 inhibitor	Phase I/Ib in solid tumors	NCT03009214	Completed
	CD44 CAR-T	Autologous T cells engineered	Phase I/II in CD44v6 positive multiple myeloma, lymphoma, stomach, breast and prostate cancer	NCT04427449	Recruiting
	CD44 CAR-T	Autologous T cells engineered	Phase I/II in breast cancer	NCT04430595	Recruiting
CD133	CD133 CAR-T	Autologous T cells engineered	Phase I in recurrent glioma	NCT03423992	Recruiting
CD166	CX-2009	CD166-directed probody drug conjugate	Phase II in advanced breast cancer	NCT04596150	Recruiting
Integrins	PF-04605412	Anti-α5β1 integrin monoclonal antibody	Phase I in advanced non-Hematologic Malignancies	NCT00915278	Terminated
	Cilengitide	Anti-αvβ3, α5β1, and αvβ5 small molecules	Phase III in glioblastoma	NCT00689221	Completed
	ProAgio	Anti-αvβ3 integrin cytotoxin	Phase I in advanced pancreatic cancer	NCT05085548	Recruiting
